# *MYCN* gene amplification is a powerful prognostic factor even in infantile neuroblastoma detected by mass screening

**DOI:** 10.1038/sj.bjc.6603149

**Published:** 2006-05-02

**Authors:** T Iehara, H Hosoi, K Akazawa, Y Matsumoto, K Yamamoto, S Suita, T Tajiri, T Kusafuka, E Hiyama, M Kaneko, F Sasaki, T Sugimoto, T Sawada, Committee of Neuroblastoma in the Japanese Society of Pediatric Oncology

**Affiliations:** 1 Department of Pediatrics, Graduate School of Medical Science, Kyoto Prefectural University of Medicine, Kawaramachi-Hirokoji Kamigyo-ku, Kyoto 602-8566, Japan; 2 Department of Medical Informatics, Niigata University Medical Hospital, Asahimachi-dori 1-754, Niigata 951-8520, Japan; 3 Saitama Children's Medical Center, Division of Hematology/Oncology, Iwatsuki, Saitama 339-0077, Japan; 4 Department of Pediatric Surgery, Graduate School of Medical Sciences, Kyushu University, Fukuoka 812-8582, Japan; 5 Department of Pediatric Surgery, Osaka University Graduate School of Medicine, Suita, Osaka 565-0871, Japan; 6 Natural Science Center for Basic Research and Development, Hiroshima University, Hiroshima 734-8551, Japan; 7 Department of Pediatric Surgery, University of Tsukuba, Tsukuba 305-0005, Japan; 8 Pediatric Surgery, Hokkaido University School of Medicine, Sapporo 060-8638, Japan

**Keywords:** neuroblastoma, infant, *MYCN*, mass screening

## Abstract

*MYCN* is the most powerful prognostic factor in cases of older children. However, how *MYCN* is related to the prognosis of infantile cases is not clear. A mass screening program was carried out by measuring urinary catecholamine metabolites (VMA and HVA) from 6-month-old infants. Of 2084 cases detected by the screening program, *MYCN* amplification (MNA) was examined by Southern blot analyses in 1533 cases from 1987 to 2000. Of the 1533 cases examined, 1500 (97.8%) showed no MNA, 20 cases (1.3%) showed MNA from three to nine copies, and 13 (0.8%) cases showed more than 10 copies. The 4-year overall survival rates of these three groups (99, 89 and 53%, respectively) were significantly different (*P*<0.001), indicating that *MYCN* copy number correlates with the prognosis. Cases with MNA more than 10 copies were more advanced than those without amplification (stage III, IV *vs* I, II, IVs; *P*<0.001). Patients with MNA more than 10 copies had significantly higher serum levels of neuron-specific-enolase (NSE) and ferritin than non-amplified patients (*P*=0.049, *P*=0.025, respectively). *MYCN* amplification was strongly correlated with a poor prognosis in infantile neuroblastoma cases. Therefore, for the selection of appropriate treatment, an accurate determination of MNA is indispensable.

Neuroblastoma (NB) is characterized by heterogeneous tumours, some of which regress spontaneously while others proliferate and progress (D'angio *et al*, 1971; Evans *et al*, 1971; Look *et al*, 1991). The prognosis for NB in infants is much more favourable than it is in older children. In 1973, the Mass Screening Program for Neuroblastoma (MSPN) was commenced for the early detection of NB in children living in Kyoto, Japan. A nationwide MSPN for 6-month-old infants began in 1985 (Sawada *et al*, 1984). The latter MSPN revealed incidences of infantile NB in the early stages and good biological prognostic factors of tumours increased (Hachitanda *et al*, 1994; Sawada *et al*, 1998). However, it has been argued that MSPN might result in the overdiagnosis of tumours, because some of the tumours might spontaneously regress (Yamamoto *et al*, 2002; Honjyo *et al*, 2003). And, the researcher has concluded that the screening was ineffective, because clustered randomized trials have not shown that screening led to a significant reduction in mortality rate from NB (Woods *et al*, 2002; Kerbl *et al*, 2003). Consequently, criticism has arisen that MSPN might detect only redundant tumours with good prognostic factors. Actually, the prognosis in most NB cases detected by MSPN has proved to be good. However, some cases detected by MSPN have poor prognostic factors resulting in relapsed disease (Kusafuka *et al*, 1995). Moreover, there are reports that the good prognosis has been obtained by early treatment in infantile NB cases with poor prognostic factors (Kusafuka *et al*, 1995; Tanaka *et al*, 1998).

Although *MYCN* is well known to be the most powerful prognostic factor in noninfantile cases of NB, how *MYCN* is related to the prognosis of infantile cases, especially those discovered by MSPN, is not clear. Therefore, we assessed *MYCN* amplification (MNA) in infantile cases. If the prognoses of infantile NB cases detected by mass screening and MNA correlate strongly, it is necessary to evaluate MNA to decide on the appropriate treatment for these cases.

## PATIENTS AND METHODS

### Analysis of urine catecholamine

Kits for screening children for urinary chatecholamines were provided to the parents at public health centres throughout Japan when they brought their child in for a health checkup at 3 months of age. Urine was collected by parents at home and sent to screening centres by mail. Urine samples were assayed for vanillylmandelic acid (VMA) and homovanillic acid (HVA) by high-performance liquid chromatography (HPLC). When children's urinary levels of either VMA or HVA were >2.5 s.d. above normal, the child was given clinical examinations for NB at a hospital. The normal range was based on levels in healthy infants of an age-matched (Sawada, 1988).

### Patient population

Between April 1987 and March 2000, the population of the target infants was 17 139 975. Of this number, 14 496 103 (84.6%) were screened for elevated catecholamine levels. Of this number, 2084 children were diagnosed as having NB based on urinary catecholamine levels and were registered with the Committee of Neuroblastoma in the Japanese Society of Pediatric Oncology.

#### Staging

The extent of the disease was evaluated according to the Evans's stage classification (Evans *et al*, 1971). The International Staging System (INSS) (Brodeur *et al*, 1993) had not yet been introduced when the MSPN began.

#### Biological features

The prognosis and clinical features of these cases were evaluated on the basis of the MNA. *MYCN* amplification in tumour samples was detected using a Southern blot analysis with *MYCN* second-exon probe according to standard procedures (Brodeur *et al*, 1984). Although cases with 10 copies or more of the *MYCN* gene are classified into the high-risk group in Japan (Kaneko *et al*, 2002), in this study the *MYCN* gene was considered amplified if there were more than three copies.

### Registry

The hospitals reported the cases to the registration centre within 2 years of the findings of elevated catecholamine levels in the screening process. The hospital reported the outcome of each case 5 years after the initial diagnosis of NB. However, the outcome of the cases diagnosed between 1999 and 2000 has been 2 years since the appearance of disease.

### Statistical analysis

The Kaplan–Meier product limit method was used to estimate the event-free survival (EFS) and overall survival (OS) from the time of diagnosis of NB. The log-rank test was performed to compare the OS probabilities between subgroups of patients. The differences between dichotomous variables were analysed by *χ*^2^ test when samples were of sufficient size. The two-tailed *t*-test was carried out to compare the distributions of continuous variables. A two-tailed *P*-value of <0.05 was considered to indicate statistical significance.

## RESULTS

Of 1533 infants with elevated urinary catecholamine levels that were examined for MNA, 33 (2.2%) had tumours with MNA. Of these 33 cases, 20 had *MNA* values from three to nine copies of the *MYCN* gene (Table 1). Seventy-seven percent of cases with no MNA had early stage (stages I, II and IVs) tumours. Thirteen cases had more than 10 copies. Of these, only 30% had early stage tumours. The cases without MNA had significantly higher percentage of early stage tumours than cases with MNA over 10 copies (*P*<0.001) (Table 2).

### Treatment and survival rates in patients with MNA

All of the 13 cases with MNA of more than 10 copies received megatherapy with stem cell transplantation and radiotherapy. Six of these cases died. None of 20 cases with MNA from three to nine copies received the megatherapy with stem cell transplantation. Sixteen of the 20 cases received mild chemotherapy, and four cases received only surgical resection without chemotherapy. Only two of the 20 cases died (Table 1). Case 15 had the unresectable tumour of stage III and died of progressive disease although he had received chemotherapy. Case 30 had the resectable tumour with *MYCN* 3 copies by the Southern blot analysis and was not classified into the high-risk group. At 3 months after the operation, this patient had relapse with bone and bone marrow metastasis and died of progressive disease. The primary tumour was judged *MYCN* amplification by the FISH method that was performed after the relapse.

### Outcome

**Figure 1:**
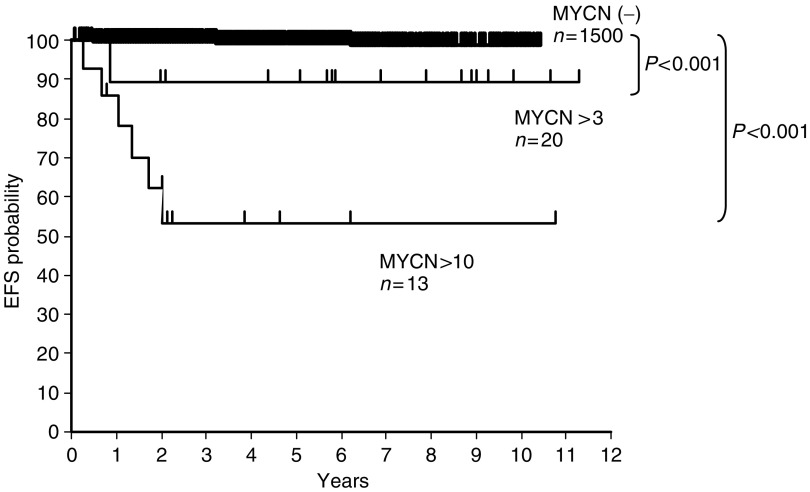
Four-year event-free survival of neuroblastoma infants detected by mass screening based on *MYCN* amplification. The curve was generated with the Kaplan and Meier product limit method. The 4-year OS rate was 99% for patients without MNA, 89% for patients with amplification from three to nine copies, and 53% for patients with more than 10 copies (*P*<0.001).

Of the 2084 cases that were detected NBs by the screening programme, only 15 cases (0.7%) died within 5 years. OS was 99%. Three-year EFS was 99% for cases without MNA (*n*=1500), 88% for cases with MNA from three to nine copies (*n*=20), and 46% for cases with MNA over 10 copies (*n*=13) (*P*<0.001) (Figure 1). The 4-year OS rate was 99% for cases without MNA, 89% for cases with MNA from three to nine copies and 53% for cases with MNA over 10 copies (*P*<0.001). In the cases with MNA over 10 copies, all of the five cases except one died of progressive disease, though they were received chemotherapy.

### Characteristics of patients with and without MNA

Table 2 lists the clinical and biological characteristics of patients with and without MNA. The cases with MNA (>10 copies) were found more frequently in advanced stages (stages III and IV), than the cases without MNA (69 and 23%, respectively; *P*<0.001). Of the cases with MNA (>10 copies), a significantly higher percentage of primary tumours was found in the adrenal glands (92%) than in those without MNA (51%; *P*=0.002). The patients with MNA(>10 copies) had significantly higher serum levels of neuron-specific enolase (NSE) and ferritin than the patients without MNA (*P*=0.049, *P*=0.025, respectively). Although the patients with MNA (>10 copies) had significantly higher urinary levels of HVA than the patients without MNA (*P*=0.008), there was no difference in urinary levels of HVA (*P*=0.985).

### Characteristics of patients with MNA

The right side of Table 2 shows clinical and biological characteristics of 33 cases with MNA more than three copies. Patients in advanced stages (stage III and IV) had significantly poorer prognoses (3-year EFS; 58.3%) than those in early stages (stage I, II and IVs) (3-year EFS; 93.3%) (*P*=0.021). The patients with primary tumours found in the adrenal gland had significantly poorer prognoses (3-year EFS; 68%) than those with the tumours at other sites (3-year EFS; 100%) (*P*=0.021). The group with high serum levels of NSE also had a significantly poorer prognosis than the group with low levels of NSE (*P*=0.0005). However, urinary levels of VMA and HVA, and serum levels of ferritin, did not correlate with clinical outcomes (*P*=0.364, 0.478 and 0.174, respectively).

## DISCUSSION

It is well known that the prognosis for NB in infants is good. Indeed, the prognosis for NB detected by the Japanese MSPN was excellent, with 98% survival. Although most of the cases detected by the MSPN had biologically favourable factors, such as no-deletion of 1p and low expression of the *TRK-A* gene, some cases with unfavourable prognostic factors have been reported (Matsunaga *et al*, 2000; Tajiri *et al*, 2001). *MYCN* is one of the most important prognostic factors in NB (Rubie *et al*, 1997; Tonini *et al*, 1997). How *MYCN* is related to the prognosis and clinical features of infantile cases, especially those discovered by MSPN, is not clear. Our large-scale study clarified the frequency and clinical features, including the prognoses, of the infantile NB cases with MNA detected that were detected by MSPN.

Among 1533 cases discovered by the MSPN, 33 cases (2.2%) showed MNA. This frequency is much lower than the 15–22% frequency of MNA cases reported in the United States and Europe (Tonini *et al*, 1997; Brodeur, 2003). In addition, in infants that were less than 1-year-old, the frequency of MNA in our study was lower than that reported in Italy (6.8%) (Tonini *et al*, 1997). This suggests that the MSPN detected a greater number of tumours that spontaneously regressed and/or matured than did the clinical examinations.

*MYCN* is the powerful prognostic factor in infants whose NB was discovered by the MSPN. The 3-year EFS rates (46%) and 4-year OS rates (53%) for patients with MNA were significantly lower than those for patients without MNA (99.3 and 99%, respectively) (*P*<0.001). According to our previous investigation, the 4-year OS rate for cases less than 12 months old with MNA of over 10 copies, which include clinically detected cases, was 41% (Ikeda *et al*, 2002). The prognosis of cases with MNA detected by MSPN might be comparatively good though prognoses cannot be compared because the researches the survival rates of cases detected clinically and cases detected by MSPN did not investigated at the same time. The infants with MNA that were detected by MSPN might be considered to have benefited from the early detection provided by the screening. Indeed, among patients with MNA, the 3-year EFS rates (93.3%) of patients in stages I, II and IVs were significantly higher than those in stages III and IV (58.3%). If these cases with MNA were not discovered in the early stage by MSPN, some malignant components of tumours would proliferate and progress. As a result, the tumours would be discovered clinically after the patients were 1-year-old. However, the number of cases with MNA is only a very small proportion (2.2%) of the total cases discovered by MSPN. In addition, it is clear that the number of NB patients increased by introduction of MSPN. Therefore, the effectiveness of MSPN discovery of patients with MNA is unclear.

Furthermore, tumours detected by MSPN might regress spontaneously (Yamamoto *et al*, 1998). Several institutions in Japan recently adopted a conservative approach (the ‘wait and see’ approach), in which children discovered to have stage I, II or IVs tumours by the MSPN were not given any therapeutic treatment in the expectation that the tumour would spontaneously regress (Yamamoto *et al*, 1998). However, a careful follow-up is necessary in cases detected by MSPN, because some of the cases were found to have MNA in the early stage. Most cases with MNA in this study did not have higher urinary VMA levels than without MNA and then, they were not predicted to have a poor clinical outcome at their initial onset. Even in the early stages (stages I, II and IVs), biopsies are required in order to determine the biological prognostic factors of the tumour.

Moreover, in this study, it became clear that patients with MNA of three to nine copies also had poor prognoses. *MYCN* gene has been analysed by the Southern blotting method for whole tumours, but this method is not able to evaluate the status MNA in individual NB cells. While, the FISH method is able to evaluate MNA individual tumour cells, however, it is difficult to determine the copy number of MNA by the FISH method. *MYCN* amplification was defined as a more than the fourfold increase of *MYCN* signals in relation to the number of chromosomes 2 in FISH method. Moreover, additional copies up to the fourfold were defined as *MYCN* gain (Spitz *et al*, 2004). Spitz reported that 6% of tumours displayed *MYCN* gain and this *MYCN* gain was associated only with a poor outcome in localized or 4s NB cases (Spitz *et al*, 2004). In our study, these patients with MNA of three to nine copies might suggest the *MYCN* gain rather than *MYCN* amplification. In cases 4 and 30, MNA were confirmed by FISH method, however, in all the cases MNA were not confirmed by it. *MYCN* amplification must be determined by adding the FISH method in these cases (Mathew *et al*, 2001).

In the studies of the USA group (COG) and the German group, the therapeutic strategy of surgical resection or observation is recommended for NB patients in stages I or II, regardless of the presence of MNA (Cohn *et al*, 1995; Kawa *et al*, 1999; Berthold and Hero, 2000; Perez *et al*, 2000). However, in Japan, patients with MNA of more than 10 copies are classified as being in a high-risk group. In the protocol for high-risk NB, patients receive intensive chemotherapy combined with stem cell transplantation (Kawa *et al*, 1999; Kaneko *et al*, 2002). Infantile NB patients with MNA as well as patients in the high-risk group more than 1-year-old with MNA of over 10 copies have been receiving intensive chemotherapy (Matsumura and Michon, 2000). In our study 29 of 33 cases with MNA received chemotherapy regardless of the stage. The use of chemotherapy might improve the prognosis of patients with MNA. In the cases with MNA over 10 copies, the treatment strategy including more intensive chemotherapy might be necessary, because five cases except one died of progressive disease. For cases with MNA, it is necessary to establish and perform the appropriate treatment, including not only surgical resection but also chemotherapy.

*MYCN* amplification was strongly and inversely correlated with the prognosis in infantile cases, although the frequency of MNA in the cases discovered through the MSPN was small (2.2%). Prediction of the presence of MNA in the tumour based on urinary levels of HVA and VMA and stage of the tumour was difficult in the cases we encountered. Our results demonstrate that evaluation of MNA is important for the selection of appropriate treatment for infantile NB.

## Figures and Tables

**Table 1:** 33 screened patients with MYCN amplification

**Case No.**	**MYCN**	**Stage**	**Surgery**	**Chemotherapy**	**Radio therapy**	**Mega therapy**	**Outcome**	**Follow-up (year)**
1	150	3	CE	VCR, CPM	(−)		NED	0.8
2	>100	4	B	VCR, CPM, VP-16, ADR, CDDP, DTI C	(−)		Tumour death	0.3^†^
3	55	4	CE	CPM, VP-16, THP-ADR, CDDP, L-PAM, CBDCA	(−)	Auto-BMT	NED	5.3
4	50	4	CE	(+)	(+) 25 gy	PBSCT	Tumour death	2.3^†^
5	50	4	CE	(+)	(−)		Tumour death	0.7^†^
6	29	4	CE	CPM, VP-16, THP-ADR, CDDP	(−)	PBSCT	Therapy complication	1.0^†^
7	24	2	CE	VCR, CPM, VP-16, THP-ADR, CDDP	(−)		NED	5.9
8	20	2	CE	CPM, VP-16, THP-ADR, CDDP	(+) 20 gy	PBSCT	Tumour death	2.7^†^
9	15	4s	CE	(+)	(−)	Auto-BMT	NED	5.1
10	14	4	B	CPM VP-16, THP-ADR CDDP → refuse	(+) 12 gy		Tumour death	2.5^†^
11	12	4	CE	(+)	(−)	CBSCT	NED	2.0
12	10	3	CE	VCR, CPM, CDDP, VP-16	(+) 10 gy		NED	3.30
13	10	4s	CE	CPM, VP16, THP-ADR, CDDP	(−)		NED	4.7
14	6	4s	CE	CPM, VP16, THP-ADR, CDDP	(−)		NED	5.0
15	5.7	3	B	VCR, CPM, VP-16, THP-ADR, CDDP	(+) 30 gy		Tumour death	0.9^†^
16	5	2	CE	VCR, CPM	(+) 24 gy		NED	10.2
17	5	2	CE	VCR, CPM, ADR, CDDP	(−)		NED	8.1
18	4–5	4	CE	VCR, CPM, THP-ADR, CDDP	(−)		NED	8.8
19	4	1	CE	VCR, CPM	(−)		NED	6.6
20	4	1	CE	VCR, CPM	(−)		NED	8.7
21	4	1	CE	VCR, CPM	(−)		NED	6.1
22	4	3	PE	CPM, VP-16, ADR, CDDP	(−)		NED	7.5
23	3.7	4s	CE	VCR, CPM, ADR, CDDP	(−)		NED	6.8
24	3	1	CE	(−)	(−)		NED	5.7
25	3	1	CE	(−)	(−)		NED	5.0
26	3	2	CE	VCR, CPM, THP-ADR, CDDP	(−)		NED	4.5
27	3	2	CE	(−)	(−)		NED	6.0
28	3	3	B	CPM, VP-16, THP-ADR, CDDP	(−)		NED	5.1
29	3	3	CE	VCR, CPM, VP-16, THP-ADR, CDDP	(−)	Auto-BMT	NED	2.1
30	3	3	CE	(−)	(−)		Tumour death	0.9^†^
31	3	4	CE	CPM, THP-ADR, CDDP	(−)		NED	8.7
32	3	4s	CE	(+)	(−)		NED	7.8
33	2–4	4	B	VCR, CPM, THP-ADR, CDDP	(−)		NED	9.7

**Table 2:** Characteristics of patients with and without *MYCN* amplification detected by mass screening for neuroblastoma

	**Number of cases (%)**					
	**MNA (>10)**	**MNA (3–9)**	**MNA (−)**			
**Patient characteristics**	**(*n*=13)**	**(*n*=20)**	**(*n*=1500)**	***P*-value**	**MNA (+, >3) 3-yr EFS**	***P*-value**
*Tumour stage*						
I	0 (0)	5 (25)	595 (40)	*P*<0.001^a^, *P*=0.05^b^	100	*P*=0.021
II	2 (15)	5 (25)	463 (31)	(1,2,4s/3,4)	86	(1,2,4s/3,4)
III	2 (15)	5 (25)	280 (19)		71	
IV	7 (54)	2 (10)	65 (4)		44	
IVs	2 (15)	3 (15)	97 (6)		100	
						
*Gender*						
Female	3 (23)	11 (55)	722 (49)	*P*=0.043^a^, *P*=0.579^b^	86	
Male	11 (77)	9 (45)	764 (51)		68	
						
*Primary site*						
Adrenal gland	12 (92)	13 (65)	764 (51)	*P*=0.002^a^, *P*=0.131^b^	68	*P*=0.021
Other abdominal	0 (0)	3 (15)	456 (30)	(adrenal gland/other site)	100	(adrenal gland/other site)
Chest	1 (8)	3 (15)	224 (15)		100	
Pelvis	0 (0)	1 (5)	50 (3)		100	
Neck	0 (0)	0 (0)	6 (0)			
						
*VMA*						
<20 *μ*g mgCr^−1^	3 (23)	4 (20)	293 (20)		100	
21–100 *μ*g mgCr^−1^	7 (54)	15 (75)	982 (67)		82	
>101 *μ*g mgCr^−1^	2 (15)	1 (5)	184 (13)		75	
	(mean: 74.6 *μ*g mgCr^−1^)	(mean: 54.8 *μ*g mgCr^−1^)	(mean: 54.4 *μ*g mgCr^−1^)	*P*=0.985^a^, *P*=0.977^b^		*P*=0.364
						
*HVA*						
<20 *μ*g mgCr^−1^	0 (0)	2 (10)	206 (14)		100	
21–100 *μ*g mgCr^−1^	7 (54)	16 (80)	1084 (74)		78	
>101 ng mgCr^−1^	6 (46)	2 (10)	170 (12)		63	
	(mean: 107.1 *μ*g mgCr^−1^)	(mean: 66.0 *μ*g mgCr^−1^)	(mean: 55.6 *μ*g mgCr^−1^)	*P*=0.008a, *P*=0.371^b^		*P*=0.478
						
*NSE*						
<15 ng ml^−1^	5 (38)	9 (45)	526 (47)		93	
16–100 ng ml^−1^	2 (15)	7 (35)	568 (51)		89	
>101 ng ml^−1^	6 (46)	2 (10)	14 (1)		25	
	(mean: 266.9 ng ml^−1^)	(mean: 32.6 ng ml^−1^)	(mean: 26.2 ng m^−1^l)	*P*=0.049^a^, *P*=0.285^b^		*P*=0.0005
						
*Ferritin*						
<30 ng ml^−1^	2 (15)	5 (25)	506 (54)		100	
31–100 ng ml^−1^	5 (38)	8 (40)	383 (41)		69	
>101 ng ml^−1^	6 (46)	1 (5)	54 (6)		43	
	(mean: 167.3 ng ml^−1^)	(mean: 55.9 ng ml^−1^)	(mean: 33.7 ng ml^−1^)	*P*=0.025^a^, P=0.032^b^		*P*=0.174

a *P*-value between MNA (>10) and MNA (−).

b *P*-value between MNA (3–9) and MNA (−).

## Appendix A1

### Participating institutions main investigators

Gunma Children's Medical Center, Gunma (Tsuchida Y, Kuroiwa M); Osaka University, Osaka (Fukuzawa M, Kusafuka T, Yoneda M); Osaka Medical Center for Maternal and Child Health, Osaka (Kawa K, Inoue M, Oue T); Kumamoto University, Kumamoto (Sera Y, Inomata Y); Tokusima University, Tokushima (Takahara H); Hyogo Children's Hospital, Hyogo (Misu H); Kyushu University, Fukuoka (Suita S, Tajiri T); Keio University, Tokyo (Yokoyama J, Morikawa Y); National Sapporo Hospital, Hokkaido (Hatae Y, Naito H); National Center for Child Health and Development (Honna T). National Nagoya Hospital, Aichi (Horibe K); Tohoku University, Miyagi (Hayashi Y); Nihon University, Tokyo (Mugishima H, Koshinaga T); Dokkyo University, Saitama (Ikeda H).
